# Role of *Porphyromonas gingivalis* gingipains in multi-species biofilm formation

**DOI:** 10.1186/s12866-014-0258-7

**Published:** 2014-10-02

**Authors:** Kai Bao, Georgios N Belibasakis, Thomas Thurnheer, Joseph Aduse-Opoku, Michael A Curtis, Nagihan Bostanci

**Affiliations:** Oral Translational Research, Institute of Oral Biology, Center of Dental Medicine, University of Zürich, Plattenstrasse 11, 8032 Zürich, Switzerland; Oral Microbiology and Immunology, Institute of Oral Biology, Center of Dental Medicine, University of Zürich, Plattenstrasse 11, 8032 Zürich, Switzerland; Barts and The London Institute of Dentistry, Queen Mary University of London, London, E1 2 AD UK

**Keywords:** Biofilm, *Porphyromonas gingivalis*, Gingipains, *Tannerella forsythia*, *Treponema denticola*, Periodontal microorganisms, Periodontal disease, Fluorescence *in situ* hybridization, Immunofluorescence

## Abstract

**Background:**

Periodontal diseases are polymicrobial diseases that cause the inflammatory destruction of the tooth-supporting (periodontal) tissues. Their initiation is attributed to the formation of subgingival biofilms that stimulate a cascade of chronic inflammatory reactions by the affected tissue. The Gram-negative anaerobes *Porphyromonas gingivalis*, *Tannerella forsythia* and *Treponema denticola* are commonly found as part of the microbiota of subgingival biofilms, and they are associated with the occurrence and severity of the disease. *P. gingivalis* expresses several virulence factors that may support its survival, regulate its communication with other species in the biofilm, or modulate the inflammatory response of the colonized host tissue. The most prominent of these virulence factors are the gingipains, which are a set of cysteine proteinases (either Arg-specific or Lys-specific). The role of gingipains in the biofilm-forming capacity of *P. gingivalis* is barely investigated. Hence, this *in vitro* study employed a biofilm model consisting of 10 “subgingival” bacterial species, incorporating either a wild-type *P. gingivalis* strain or its derivative Lys-gingipain and Arg-gingipan isogenic mutants, in order to evaluate quantitative and qualitative changes in biofilm composition.

**Results:**

Following 64 h of biofilm growth, the levels of all 10 species were quantified by fluorescence *in situ* hybridization or immunofluorescence. The wild-type and the two gingipain-deficient *P. gingivalis* strains exhibited similar growth in their corresponding biofilms. Among the remaining nine species, only the numbers of *T. forsythia* were significantly reduced, and only when the Lys-gingipain mutant was present in the biofilm. When evaluating the structure of the biofilm by confocal laser scanning microscopy, the most prominent observation was a shift in the spatial arrangement of *T. denticola*, in the presence of *P. gingivalis* Arg-gingipain mutant.

**Conclusions:**

The gingipains of *P. gingivalis* may qualitatively and quantitatively affect composition of polymicrobial biofilms. The present experimental model reveals interdependency between the gingipains of *P. gingivalis* and *T. forsythia* or *T. denticola*.

## Background

Periodontal infections, or periodontal diseases, are a set of chronic inflammatory diseases that destroy the tooth-supporting (periodontal) tissues. They are caused by oral bacterial biofilms attaching on the tooth surface. They have the capacity to trigger a series of inflammatory responses, which may destroy the gingival tissue and the alveolar bone supporting the tooth, if they become exacerbated [1,2]. With regards to its capacity as an ecological niche, the oral cavity can be colonized by more than 700 species [3] and approximately 500 of those can be present within the forming biofilms [4,5]. Among the biofilm-associated microbiota, earlier clinical epidemiological studies have demonstrated that three species in particular, also designated as the “red complex”, are more associated with periodontal disease than others. These are namely *Porphyromonas gingivalis*, *Tannerella forsythia*, and *Treponema denticola*. They are all Gram-negative anaerobes, with a high proteolytic activity [6]. Among these three*, P. gingivalis* holds a prominent role in orchestrating the virulence of the biofilm and the consequent tissue inflammatory response, earning itself the characteristics of a “keystone” periodontal pathogen [7,8]. *P. gingivalis* expresses several virulence factors, including, fimbriae, LPS, and its cysteine proteases, namely gingipains [9]. These include the arginine-specific proteinases RgpA and RgpB, and the lysine-specific proteinase Kgp, which represent the majority of the cell-surface proteinases of *P. gingivalis* [10]. Clinical studies have demonstrated that periodontal infection associated with *P. gingivalis* can result in significantly elevated systemic antibody response to the gingipains [11,12].

When growing in a subgingival (below the gingival margin) biofilm under strict anaerobic conditions, *P. gingivalis* is highly dependent on its gingipains for utilizing free amino acids as a source of carbon and nitrogen [13]. Moreover, unlike other gram-negative bacteria, *P. gingivalis* does not produce siderophores to sequester and transport iron but its gingipains mediate the uptake of iron from hemoglobin, heme proteins, and ferritin [14,15]. Gingipains are also considered important in the capacity of *P. gingivalis* to evade host defences, by degrading antibacterial peptides, such as neutrophil-derived α-defensins, complement factor, such as C3 and C4, T cell receptors, such as CD4 and CD8 [16]. Alternatively, *P. gingivalis* and its gingipains can subvert the host immune response by proactively manipulating host molecules, particularly of the complement [17,18]. For instance, *P. gingivalis* may perturb the cross-talk between C5a receptor and toll-like receptor signalling in order to prevent bacterial clearance and cause dysbiosis [19], eventually resulting in periodontal bone loss [20,21]. The construction and phenotypic analysis of isogenic protease mutants of *P. gingivalis* have confirmed putative functions for these proteolytic enzymes [22]. *In vivo* studies using the *P. gingivalis* mutant strains in animal models have reinforced the view that the gingipains can modulate the infection process [23-26]. *In vitro* studies have demonstrated an involvement of the gingipains in the regulation of inflammatory mediators from various host cells, including IL-1 α, IL-1β, IL-18 [27], receptor activator of NF-κB ligand (RANKL) [28-31], tumor necrosis factor-α converting enzyme (TACE) [32], protease-activated receptor (PAR)-2 [33], or soluble triggering receptor expressed on myeloid cells (sTREM)-1 [34].

Understanding how different organisms act within a given polymicrobial biofilm brings us closer to understanding the etiological mechanisms of periodontal disease [1]. That is because interactions among different bacterial cells can determine the structural characteristics, maturation and virulence of the biofilms [35-37]. These interactions can occur at several levels, including physical contact, metabolic exchange, and signal-mediated communications [38]. Additionally, species-specific virulence factors may regulate bacterial growth, hence altering the conditions of the ecological niche for biofilm formation. In this respect, most studies involving gingpains have focused on *P. gingivalis* as a single species, which might overlook the bacterial interactions within a complex biofilm community. Therefore, the present study used a 10-species “subgingival” biofilm, aiming to investigate the role of gingipains on the growth and structure of the biofilm, by incorporating *P.* g*ingivalis* gingipain-deficient strains.

## Results

### Quantitative evaluation of bacteria in the biofilm

**Figure 1:**
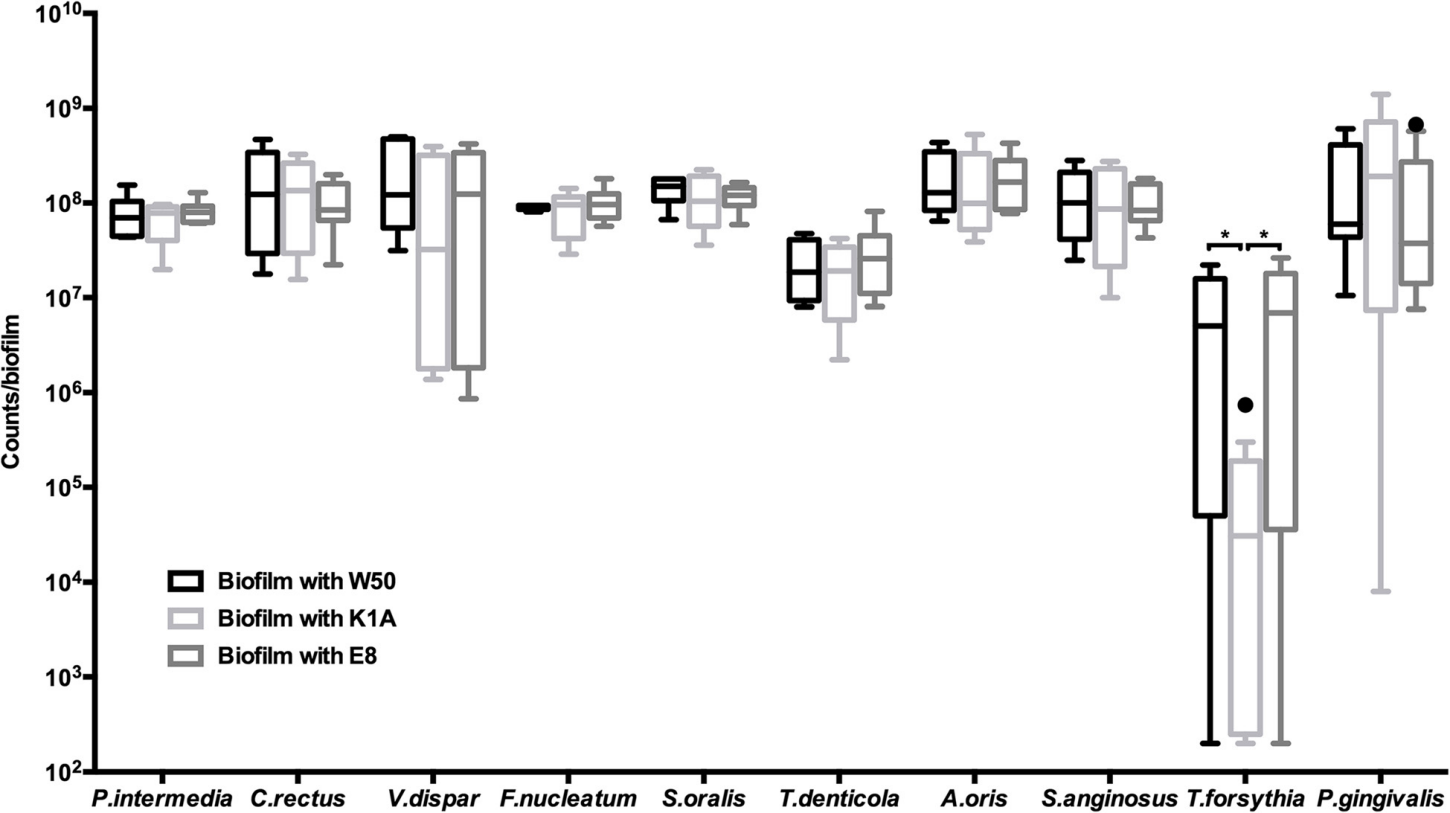
**Bacterial numbers of each species in the biofilms.** Numbers of each strain were counted by epifluorescence microscopy, following staining by FISH or IF. Data was plotted on a logarithmic scale. Asterisk (*) indicates significant differences (*P ≤ 0.01*) between the groups. Open circle indicates data points considered as outliers. Groups are defined by the use of the corresponding *P. gingivalis* strain (W50; wild-type, E8; Arg-gingipain-deficient mutant, K1A; Lys-gingipain-deficient mutant).

The numbers for each individual species within the different biofilm groups were quantified either by fluorescence *in situ* hybridization (FISH) or by immunofluorescence (IF). The growth of *P. gingivalis* was not affected depending on whether the wild-type or the gingipain-deficient strains were used. Statistically, compared to the wild-type strain, the *P. gingivalis* gingipain-deficient strains did not cause significant changes in the growth of the remaining nine-biofilm species in the biofilm, with the exception of *T. forsythia* (Figure 1). In particular, the presence of the Lys-gingipain deficient strain K1A caused a significant (*P* < 0.01) reduction of *T. forsythia* cell numbers, compared to the wild-type W50, or the Arg-gingipain-deficient strain E8 (29.9-fold and 38.6-fold, respectively). However, no significant differences in *T. forsythia* numbers were observed between the wild-type W50 and the Arg-gingipain-deficient E8 biofilm groups.

### Qualitative evaluation of biofilm structure by confocal microscopy

**Figure 2:**
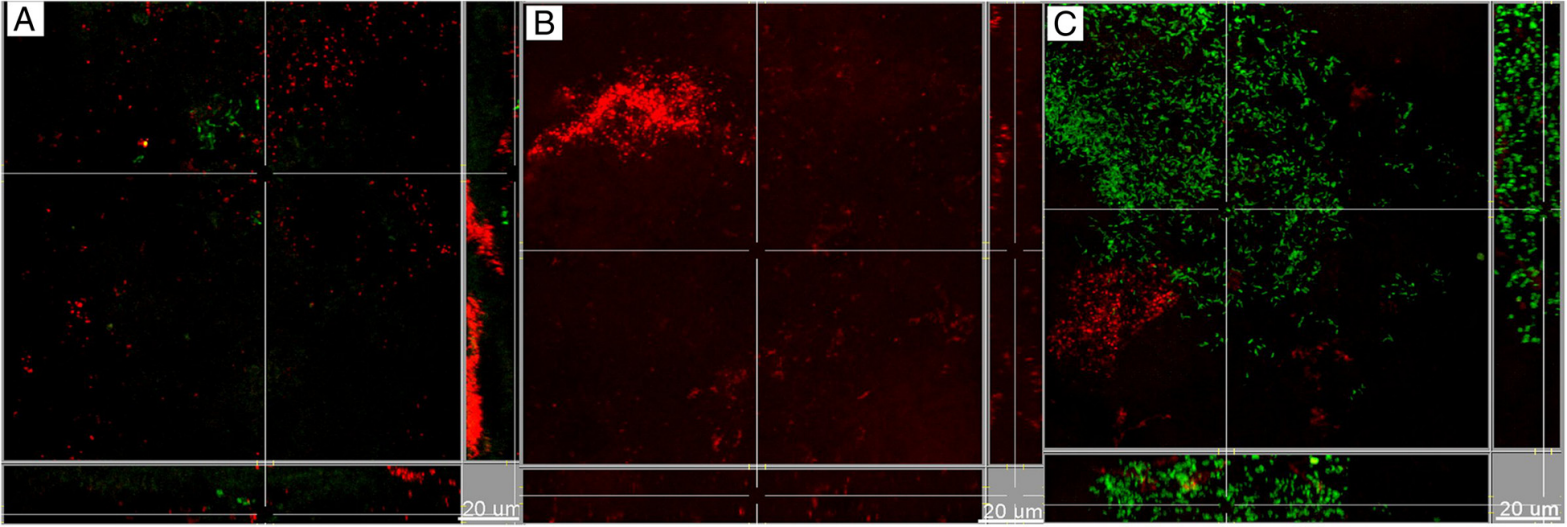
**Localization of** ***P. gingivalis*** **and** ***T. forsythia*** **within the biofilms.** Multiplex IF staining was performed for P. gingivalis (red) and T. forsythia (green). Groups are defined by the use of the **(A)** wild-type, **(B)** Arg-gingipain-deficient mutant, **(C)** Lys-gingipain-deficient mutant *P. gingivalis* strain in the biofilm. Scale bar length: 20 μm.

Having identified that a dependency exists between the Lys-gingipain and the growth of *T. forsythia*, we further investigated the structure of the biofilm by means of confocal laser scanning microscopy (CLSM), and evaluated changes in the presence of the *P. gingivalis* gingipain-deficient strains. Firstly, the focus was placed on the structural association or localization between *P. gingivalis* and *T. forsythia*. Within the biofilm structure, *P. gingivalis* appeared in variable size aggregates or clusters of its own species, with no marked differences observed between the wild-type W50 and the gingipain-deficient strains (Figure 2). The distribution pattern of *T. forsythia* was in more scattered clusters, observed often in the immediate vicinity of *P. gingivalis* clusters, but not strongly intertwining each other (Figure 2). This pattern was observable irrespective of the use of *P. gingivalis* wild-type W50 or the Arg-gingipain deficient strain E8, whereas when the Lys-gingipain deficient strain K1A was included in the biofilm instead, this association was less obvious (Figure 2), presumably due of the low *T. forsythia* numbers.

**Figure 3:**
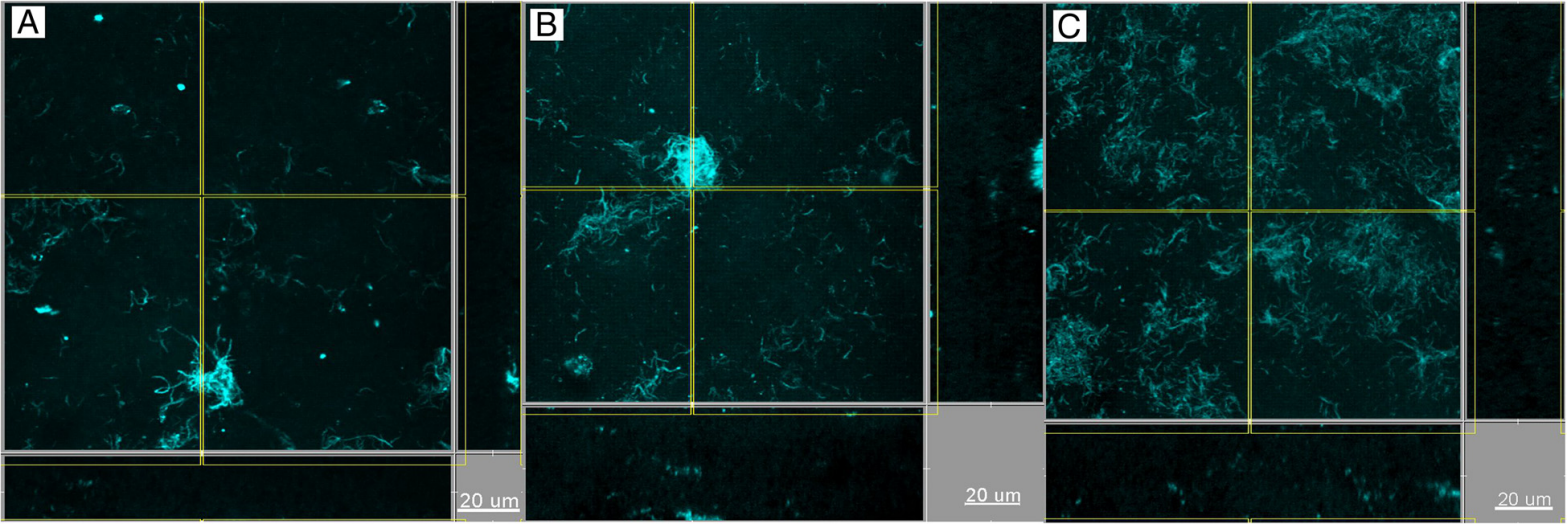
**Localization of** ***T. denticola*** **within the biofilms.** IF staining was performed for *T. denticola* (cyan). Groups are defined by the use of the **(A)** wild-type, **(B)** Arg-gingipaindeficient mutant, **(C)** Lys-gingipain-deficient mutant, *P. gingivalis* strain in the biofilm. Scale bar length: 20 μm.

**Figure 4:**
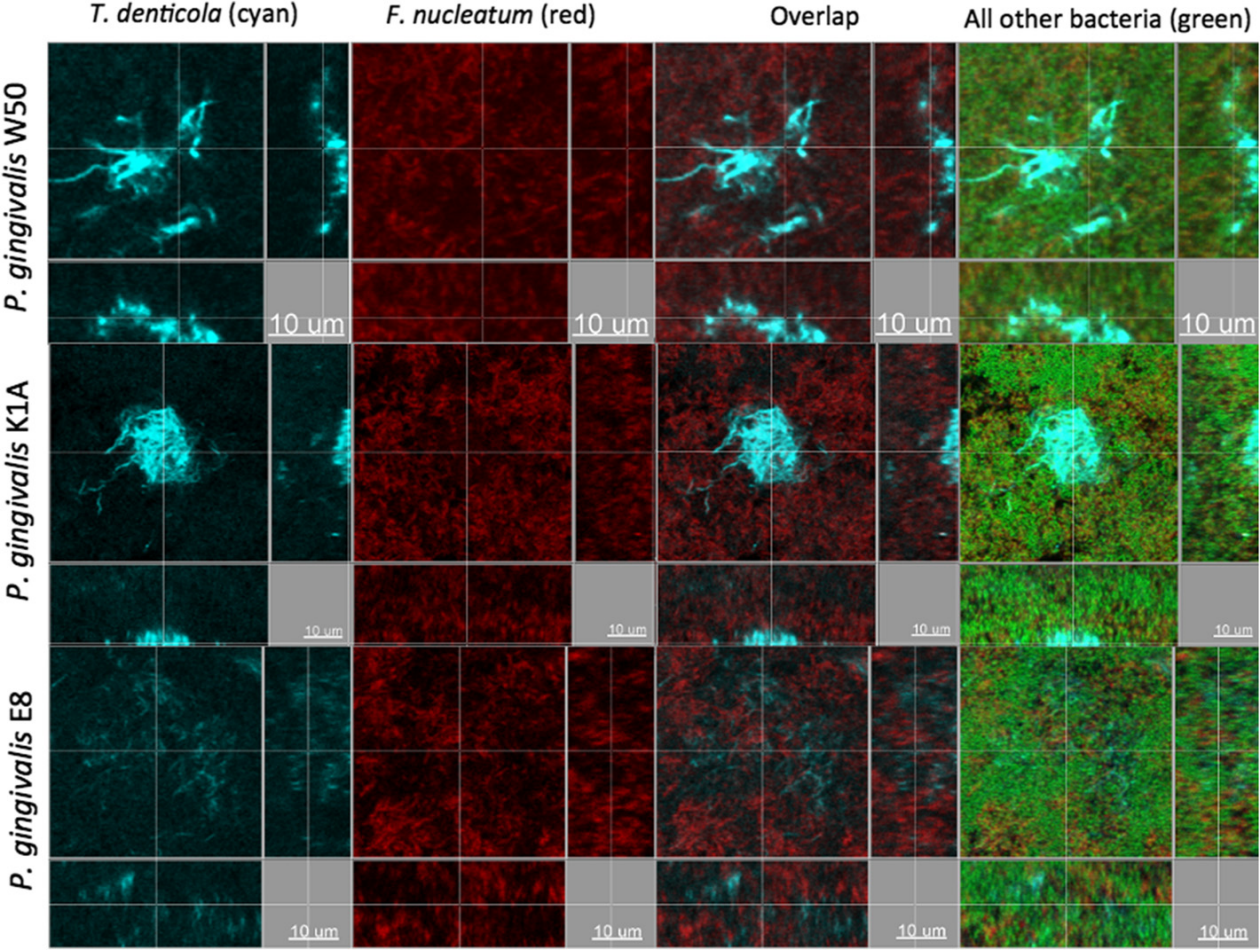
**Localization of** ***P. gingivalis, F. nucleatum*** **and** ***T. denticola*** **within the biofilms.** IF staining was performed for *T. denticola* (cyan), *F. nucleatum* (red) and YoPro-1 iodide & Sytox Green mixture for all other bacteria (green). Groups are defined by the use of the corresponding *P. gingivalis* strain (W50; wild-type, E8; Arg-gingipain-deficient mutant, K1A; Lys-gingipain-deficient mutant) in the biofilm. Scale bar length: 20 μm.

It was of further interest to investigate the localization of *T. denticola* within the biofilm structure, as the third member of the “red complex” cluster. Interestingly, *T. denticola* formed aggregates or clusters in the presence of the *P. gingivalis* wild-type strain W50, as was the case also when the Lys-gingipain deficient strain K1A was used. However, in the presence of the Arg-gingipain deficient strain E8, *T. denticola* lost this “cluster-like” conformation in the biofilm, and acquired a more even and “thread-like” distribution (Figures 3 and 4). *Fusobacterium nucleatum* was also strongly present throughout the biofilm and appeared to be evenly distributed among these *T. denticola* structures (Figure 4).

## Discussion

As it is well established that periodontal diseases are initiated by a mixed-species biofilm [39,40], *in vitro* biofilm models, may be more accurate in studying the causative factor of the disease, than single species in planktonic form [37,41,42]. The present study investigated the involvement of *P. gingivalis* gingipains in the quantitative and qualitative composition of a polymicrobial biofilm consisting of 10 species that are frequently comprising part of the subginvival microbial flora. Among their many properties, gingipains are important for the growth of *P. gingivalis* and as transporters for iron [14]. While in planktonic culture *P. gingivalis* gingipain deficient strains require longer doubling times [43], their incorporation into a polymicrobial biofilm did not yield differences in numbers, compared to the wild-type strain. Hence, the growth characteristics of *P. gingivalis* may differ depending on whether it grows in planktonic or biofilm state. When present in a biofilm, gingipains do not appear to be crucial for the growth of *P. gingivalis*, as shown here. Interestingly, among the remaining nine species in the biofilm, the only one whose growth was affected by the presence of gingipains was *T. forsythia*. In particular, the *P. gingivalis* Lys-gingipain deficient strain resulted in a strong reduction in *T. forsythia* numbers after 64 h of biofilm growth. Reversely, this indicates that the Lys-gingipain produced by *P. gingivalis* has an additive effect on the growth of *T. forsythia* in the biofilm. This denotes a synergistic association between *T. forsythia* and *P. gingivalis* as mutual components of a polymicrobial community, which is mediated by the Lys-gingipain of the latter.

Previous studies have shown that gingipains are crucial for the co-aggregation of *P. gingivalis* or its co-adhesion with other species, such as *T. denticola* [44-46], or for the invasion of host cells [47]. Hence, the gingipains may not only affect the quantitative composition but also the structural conformation of the biofilm. For this reason, the biofilm architecture was also investigated by CLSM. *P. gingivalis* occurred in distinguishable and evenly distributed clusters within the biofilm regardless of whether it expressed a gingipain or not. The communities of *T. forsythia* within the biofilm exhibited similar patterns to those of *P. gingivalis*, and were frequently co-localized, yet without impinging onto each other. The proximal association of these two species’ communities in biofilm may hint for an ecological relationship. This is also substantiated by the notable absence of *T. forsythia* clusters from the vicinity of the Lys-gingipain deficient *P. gingivalis*. Hence, this gingipain may be important for the growth of *T. forsythia* and its spatial interdependency to *P. gingivalis* within the biofilm. This observation could represent an example of the metabolic responses and bacterial quorum-sensing within the biofilm [48].

Another interesting observation of the present study is that of the structural re-arrangement of *T. denticola* in the biofilm, depending on the presence or absence of the Arg-gingipain. Earlier studies have shown that other species can interact with *P. gingivalis* in both planktonic suspensions and biofilms [46,49,50]. A recent study using the similar multi-species biofilm model as here demonstrated that *P. gingivalis* and *T. denticola* have the tendency to co-colonize gingival epithelial tissue [51]. In a dual *P. gingivalis - T. denticola* biofilm, it was also demonstrated that gingipains do contribute to their interaction [50]. In the present experimental model, *T. denticola* cells formed dense circular clumps with the wild-type *P. gingivalis* strain. However, in the presence of the *P. gingivalis* Arg-gingipain deficient strain, this conformation was lost and *T. denticola* cells were instead arranged in looser threaded structures, even though their numbers in the biofilm were not changed. This finding provides further evidence of the ecological association between *P. gingivalis* gingipains and the structural arrangement of *T. denticola* in the biofilm. It is difficult at this stage to interpret the biological meaning of this change in *T. denticola* structure. Of note, in a recent study using the similar biofilm model it was demonstrated that omission of streptococci from the biofilm resulted in numeric changes of *P. gingivalis* and *P. intermedia*. The latter also lost its aggregated form and was arranged in filamentous long chains, resembling those of the missing streptococci [35].

## Conclusions

This study showed that the gingipains of *P. gingivalis* promote quantitative and qualitative shifts in the composition and structure of a multi-species biofilm. More specifically, the Lys-gingipain enhances the growth of *T. forsythia*, whereas the Arg-gingipain promotes the aggregation of *T. denticola* in the biofilm. These ecological interactions are interpreted as synergistic ones, and may support the survival and the virulence of the biofilm community as a whole.

## Methods

### *In vitro* biofilm formation

The method used to develop 10 species biofilm is a modification of a previous report of this model [52], with major changes described below. The following strains were used in this study: *Prevotella intermedia* ATCC 25611 T (OMZ 278), *Campylobacter rectus* (OMZ 398), *Veillonella dispar* ATCC 17748 T (OMZ 493), *Fusobacterium nucleatum subsp. nucleatum* (OMZ 598), *Streptococcus oralis* SK248 (OMZ 607), *T. denticola* ATCC 35405 T (OMZ 661), *Actinomyces oris* (OMZ 745), *Streptococcus anginosus* ATCC 9895 (OMZ 871), *T. forsythia* (OMZ 1047), *P. gingivalis* W50 (OMZ 308), *P. gingivalis* K1A (OMZ 1126) and *P. gingivalis* E8 (OMZ 1127). The latter two are genetically modified strains of *P. gingivalis* W50, with a deletion of Lysine-gingipain (*kgp*) and Arginine-gingipain (*rgpArgpB*) genes, respectively [22]. Each of the biofilm groups in this experimental design contains one of the three *P. gingivalis* strains and all other 9 species. For biofilm formation, 200 μl of bacterial cell suspension, containing equal volumes and densities (OD_550_ = 1.0) of each strain were added onto pellicle-coated hydroxyapatite discs (diameter 5 mm), in 1.6 ml growth medium supplemented with 0.5% hemin, as described earlier [53]. The medium was renewed after 16 h and 24 h, during the total incubation time of 64 h. The discs were dip-washed three-times daily.

### Biofilm harvesting

After 64 h of incubation, the biofilm discs were ready to be harvested. For quantification of the bacterial numbers in the biofilm, the discs were vigorously vortexed for 2 min in 0.9%NaCl and then sonicated at 25 W in a Sonifier B-12 (Branson Sonic Power Company) for 5 sec. For confocal laser scanning microscopy (CLSM) of the biofilm structure, the discs were dip-washed and immediately fixed in 4% paraformaldehyde (Merck, Darmstadt, Germany) at 4°C for 1 h before being processed for fluorescence *in situ* hybridization (FISH) or immunofluorescence (IF) analysis.

### Quantification of bacteria by FISH and IF

The bacterial suspensions were diluted, fixed on the slides, stained and counted as described [54,55]. For FISH staining, slides were fixed at 4°C with 4% paraformaldehyde in PBS for 20 min and for IF staining they were fixed at room temperature with methanol for 2 min, before they were incubated with the antibodies at 37°C. FISH was used for the evaluation of *S. oralis*, *S. anginosus* and *V. dispar* (oligonucleotide probes listed in Table 1), while IF was used for the evaluation of *T. denticola*, *C. rectus*, *T. forsythia*, *P. gingivalis*, *P. intermedia*, *F. nucleatum* and *A. oris* (antibodies listed in Table 2).

**Table 1:** **16S rRNA oligonucleotide probes for FISH**

**Target**	**Probe name**	**FA**	**Sequence (5’ → 3’)**	**Ref.**
*V. dispar*	VEI217	45%	AATCCCCTCCTTCAGTGA	[55]
*S. oralis*	MIT447	25%	CACYCGTTCTTCTCTTACA	[56]
*S. anginosus*	Sang1203	45%	GGTACACCTTCACCACAC	[57]

FA; Formamide concentration in the hybridization buffer.

**Table 2:** **Antibodies for IF**

**Target**	**Antibody name**	**Isotype**	**Ref.**
*C. rectus*	212WR2	mouse IgG3	[58]
*T. forsythia*	103BF1.1	mouse IgG2b	[59]
*P. gingivalis*	61BG1.3	mouse IgG1	[60]
*P. intermedia*	37BI6.1	rat IgG2b	[53]
*F. nucleatum*	305FN1.2	mouse IgM	[61]
*A. oris*	396AN1	mouse IgM	[61]
*T. denticola*	CD-1	Rabbit polyclonal antiserum	[41]

For FISH, the fixed samples were first pre-hybridized, with hybridization buffer containing 0.9 M NaCl, 20 mM Tris/HCl (pH 7.5), 0.01%SDS, formamide (as indicated in Table 1) at 46°C, for 15 min. Following this step, hybridization was performed using specific oligonucleotide probes (Table 1) at the same temperature, for 3 h. Thereafter, the samples were incubated at 48°C with pre-warmed wash buffer containing 20 mM Tris/HCl (pH7.5), 5 mM EDTA, 0.01% SDS, and 40–159 mM NaCl for 30 min. For CLSM and image analysis, the samples were counterstained with a mixture of 3 μM YoPro-1 iodide (Invitrogen, Basel, Switzerland) and 15 μM Sytox Green (Invitrogen, Basel, Switzerland) then embedded with 10 μl Mowiol [55] with the biofilm surface facing towards the chamber slides. Prior to qualification, the samples were coated with mounting buffer consisting of 90% ultrapure glycerol and 10% 25 mg/g DABCO (Sigma-Aldrich, Buchs, Switzerland), on 24 well slides, Finally, the stained bacterial cells were visualized under an Olympus BX60 fluorescence microscope (Olympus Optical AG, Volketswil, Switzerland), at 100× magnification.

The box-plot data presented derives from four independent experiments each performed in triplicate biofilm cultures. The values were logarithmically transformed, and then inserted to Prism v.6 software (GraphPad, La Jolla California USA). The statistical differences between the groups were calculated by one-way ANOVA, using the Tukey’s post-hoc test for multiple comparisons (*P* ≤ 0.01).

### Confocal laser scanning microscopy and image analysis

For evaluation of the biofilm structure, CLSM was used for each one of the four independent experiments. The biofilm-containing discs stained by FISH or IF were visualized using a Leica SP-5 microscope at the Center of Microscopy and Image Analysis of the University of Zürich (ZMB), with a resonant scanner system (8000 Hz), a diode laser (405 nm excitation), an argon laser (458 nm/476 nm/488 nm/496 nm/514 nm excitation) and a helium neon laser (561 nm/594 nm/633 nm excitation). Filters were set to 500–540 nm, 570–630 nm, and 660–710 for detection of YoPro-1 iodide & Sytox Green mixture, Cy3 and Cy5, respectively. All images were captured using a 63 × objective (glycerol immersion, NA 1.3). Stacked images were further processed using the Imaris™ 7.4.0 software (Bitplane, Zürich, Switzerland), in order to virtually reconstruct the biofilm structure.
